# Pharmacologic disruption of Polycomb Repressive Complex 2 inhibits tumorigenicity and tumor progression in prostate cancer

**DOI:** 10.1186/1476-4598-10-40

**Published:** 2011-04-18

**Authors:** Francesco Crea, Elaine M Hurt, Lesley A Mathews, Stephanie M Cabarcas, Lei Sun, Victor E Marquez, Romano Danesi, William L Farrar

**Affiliations:** 1 Cancer Stem Cell Section, Laboratory of Cancer Prevention, National Cancer Institute at Frederick, Center for Cancer Research, National Cancer Institute, Frederick, MD, USA; 2 MedImmune, Gaithersburg, Maryland 20878, USA; 3 Chemical Biology Laboratory, National Cancer Institute at Frederick, Center for Cancer Research, National Cancer Institute, Frederick, MD, USA; 4 Department of Internal Medicine, Division of Pharmacology, Pisa Medical School, Pisa, Italy

## Abstract

**Background:**

Polycomb repressive complex 2 (PRC2) mediates gene silencing through histone H3K27 methylation. PRC2 components are over-expressed in metastatic prostate cancer (PC), and are required for cancer stem cell (CSC) self-renewal. 3-Dezaneplanocin-A (DZNeP) is an inhibitor of PRC2 with broad anticancer activity.

**Method:**

we investigated the effects of DZNeP on cell proliferation, tumorigenicity and invasive potential of PC cell lines (LNCaP and DU145).

**Results:**

Exploring GEO and Oncomine databases, we found that specific PRC2 genes (EED, EZH2, SUZ12) predict poor prognosis in PC. Non-toxic DZNeP concentrations completely eradicated LNCaP and DU145 prostatosphere formation, and significantly reduced the expression of CSC markers. At comparable doses, other epigenetic drugs were not able to eradicate CSCs. DZNeP was also able to reduce PC cell invasion. Cells pre-treated with DZNeP were significantly less tumorigenic (LNCaP) and formed smaller tumors (DU145) in immunocompromised mice.

**Conclusion:**

DZNeP is effective both in vitro and in vivo against PC cells. DZNeP antitumor activity is in part mediated by inhibition of CSC tumorigenic potential.

## Introduction

Prostate cancer (PC) is the second leading cause of cancer death in men in the US [1]. Disease confined to the prostate is curable, while metastatic PC is associated with poor prognosis. Although endocrine therapy and docetaxel improve patient survival, metastatic disease eventually leads to death [2]. Thus, the identification of new drugs to target PC progression and metastasis is highly warranted.

In the past few years, it has been determined that PC contains a cancer stem cell (CSC)-compartment [3]. This compartment shares with normal stem cells an unlimited potential for self-renewal and the ability to differentiate in many cell types. When injected into immunocompromised mice, CSCs are highly tumorigenic cells compared to the bulk population [4] and can be as rare as 0.1% of the total tumor mass. CSCs are considered the seeds of tumor progression, metastasis and recurrence [5]. In addition, they are resistant to conventional therapy. Thus, the identification of targets that specifically inhibit CSC growth may improve PC patient survival [6]. Traditionally, CSC have been identified by two methods: *in vitro *culture of spheres in serum-replacement medium [7], and isolation of tumorigenic cells based on the expression of specific cell surface markers [4]. Our group identified CD44^+^/24^- ^cells as the tumor-initiating fraction in LNCaP and DU145 cell lines [4]. Duhagon et al. [8], and Dubrovska et al. [7] demonstrate that cells cultured in serum-replacement medium supplied with specific growth factors are highly tumorigenic and express several CSC markers. An additional method to test "stemness" features in cancer cells is the ability to become locally invasive through a structural change termed epithelial-to-mesenchymal transition (EMT) [9]. EMT is also a model used to investigate the metastatic potential of cancer cells [10]. Interestingly, CSCs in PC share all these three characteristics: CD44^+^/24^- ^cells are highly tumorigenic, give rise to anchorage-independent growth in serum-replacement medium[4] and are more invasive [11].

CSCs are characterized by the expression of several stem cell-specific genes, including *nanog, oct3-4 and c-myc *[6]. Among these, Polycomb Repressive Complexes (PRCs) play a crucial role. Polycomb genes are organized in multimeric complexes that mediate specific histone post-translational modifications and gene silencing [12]. During development, PRCs orchestrate body segmentation and tissue specification. PRC2 mediates histone H3 lysine 27 trimethylation, thereby silencing lineage-specific genes and maintaining stem cell pluripotency. In PC cells, PRC2 genes are over-expressed in the CD44^+^/24^- ^fraction[4], and are required for anchorage independent growth[13]. In addition, Polycomb genes orchestrate metastasis-suppressor gene silencing during EMT [14,15]and PC chemo-resistance[16]. In particular, The PRC2 component EZH2 is predictive of shorter disease progression and poor treatment outcome in PC patients [17]. Thus, PRC2 could be a viable target to deplete CSCs, counter metastatic spreading and improve patient survival.

3-Dezaneplanocin-A (DZNeP) is an S-adenosyl-L-homocysteine hydrolase inhibitor first tested against Ebola virus [18]. More recently, this compound showed a broad anticancer activity, with little or no effects on non-transformed cells [19]. DZNeP inhibits EZH2 histone methyltransferase activity, and induces protein degradation of PRC2 components (EZH2, EED, SUZ12). DZNeP-dependent histone demethylation reactivates a set of PRC2-silenced genes in cancer cells, thereby causing apoptosis. Recently, DZNeP was shown to be effective against brain cancer stem cells, and to inhibit *in vivo *glioblastoma formation [20].

Due to the widespread role of PRC2 genes in PC tumorigenicity progression and invasion, we sought to determine whether DZNeP is active against PC CSCs. To test this hypothesis, we treated two PC cell lines with DZNeP, specifically investigating the effects on CSC markers, prostatosphere formation and EMT. We also carried out *in vivo *experiments to test the hypothesis that DZNeP impairs CSC tumorigenic potential. In addition, we queried patient databases to investigate the role of PRC2 genes and PRC2 targets in PC prognosis, as well as to dissect viable pathways modulated by DZNeP in PC cells.

## Materials and methods

### Cell culture

The PC cell lines LNCaP and DU145 were obtained from American Type Culture Collection (Manassas, VA). LNCaP cells are derived from a lymph node metastasis and DU145 cells from a brain metastasis. Both cell lines are derived from androgen-independent prostate cancers. LNCaP still express the androgen receptor (AR) and a wild-type p53 gene, DU145 are androgen receptor negative and p53-mutated. LNCaP and DU145 were maintained in culture medium (RPMI-1640 and DMEM, respectively) with 10% fetal bovine serum, glutamine (1%), and penicillin-streptomycin (1%). DZNeP was dissolved in water and diluted in culture medium immediately before use. Trichostatin A (TSA) and 5-aza-2-deoxycitidine were dissolved in dimethyl sulfoxide (DMSO) and diluted in culture medium immediately before use. Final DMSO concentration never exceeded 0.1%. The same concentration of DMSO was used as a control for these experiments.

### Cell cycle and apoptosis analysis

Cells were seeded at 50% confluence to ensure logarithmic growth and treated with 1 μM DZNeP for 3 and 5 days. This concentration is not harmful for non-transformed cells[19], although it showed anti-tumor activity. Following treatment, one million cells were fixed in ice cold 70% ethanol overnight. Following fixation, cells were centrifuged and resuspended in PBS containing 40 μg/mL propidium iodide and 100 μg/mL RNAse A and incubated at 37°C for 1 hour. We did not observe cell cycle distribution differences for a 3 day treatment. Thus, only the effects after 5 day treatment will be discussed. Apoptosis assays were performed as previously described [21]. Apoptosis was also measured after treatment with 5-aza (0.5 μM, 3d) and TSA (20ng/ml, 3d). These concentrations were chosen because they are not harmful for normal cells[22,23], thus they are comparable to the DZNeP dose we employed.

### Prostatosphere formation assay

Prostatospheres were generated according to the protocol described by Duhagon et al. [8]. Spheres number and volume were evaluated through GelCountTM automatic plate scanner (Oxford Optronics) and GelCount Version 0.025.1 software (Oxford Optronics).

### Western Blot

Total protein was isolated from LNCaP and DU145 cells using RIPA lysis buffer (Thermo Scientific, Waltham, MA USA) and quantified using the BCA protein assay kit (Pierce) kit. Thirty μg of protein extract was loaded per lane into a 4% to 20% Tris-glycine gel (Invitrogen, Carlsbad, CA). Proteins were transferred to a polyvinylidene fluoride membrane, blocked in 10% nonfat dry milk, 0.1% Tween-20 PBS, incubated with primary (anti-EZH2, Millipore) and secondary (LI-COR Biosciences, Lincoln Nebraska USA) antibodies, and scanned by the LI-COR Odyssey IR Imaging System as previously described [4].

### Cytofluorimetric Assay

Flow-cytometric discrimination, based on CD44 and CD24 expression, was performed as previously described [4].

### Matrigel invasion assay

Matrigel assays measure the ability of cancer cells to invade through a protein matrix. This is considered an *in vitro *model for early metastatic stages, namely basal membrane invasion. We performed this assay as previously described [24]. For experiments involving isolation of top 'non-invading' and bottom 'invading' cells, parallel invasion chambers were setup. For non-invading cells, the bottom of the membrane was scrubbed with a cotton swab and cells on top were harvested using 500 μl of trypsin incubated at 37°C for 5 minutes. To obtain the invading cells, the top of the membrane was scrubbed with a cotton swab and the chambers were placed into another 24-well plate containing 500 μL of trypsin incubated at 37°C for 5 minutes. RNA was extracted from invading and non-invading cells using the Trizol reagent. cDNA was prepared and measured by quantitative PCR, as described[11]. An EZH2 TaqMan gene expression assay (Applied Biosystems) was employed for this purpose. This experiment was not repeated, due to the very low yield of RNA extraction for invading cells.

### Gene expression assay

DU145 cells were treated with DZNeP 10 μM, 3d. At the end of the treatment, RNA was extracted and retro-transcribed, as described in the previous paragraph. Gene expression level was determined for the following genes, involved in EMT, through validated Applied Biosystems assays: CDH1, FGF2, MMP3, c-MYC, SNAIL, TGFB1, TWIST, FGF9, NRG1, TGBR2, SPOCK3. 18S RNA was employed as a reference, and relative expression was quantified by 2^-ΔΔCt^method. We reported only changes greater than 2-fold.

### *In vivo *experiments

Animal Care was provided in accordance with the procedures outlined in the 'Guide for Care and Use of Laboratory Animals' (Institute of Laboratory Animal Resources, 1996). LNCaP and DU145 cells were treated with 1 or 10 μM DZNeP for three days. After three days, cells were trypsinized, washed once with PBS, then resuspended in serum-replacement medium at 1000 viable cells per 50 μl, mixed with an equal volume of matrigel (BD Biosciences) and injected subcutaneous into male NOD/SCID (non obese diabetic, severe combined immunodeficiency) mice (Jackson Labs, Bar Harbor, ME, USA). For controls, 10^6 ^(DU145) and 5*10^6 ^(LNCaP) viable cells per animal were injected (8 animals per treatment group). Cell viability was assessed upon trypsination through Trypan Blue staining. Untreated cells were plated at the same time and injected into 6 NOD/SCID mice following the same procedure. Mice were monitored daily for palpable tumour formation for a total of 45 days. Tumour size was measured with calipers. Tumour volume was determined by the following formula: V = W^2^XL/2 [25]. Once tumours reached greater than 1.5 mm in one dimension, mice were euthanized and tumours were collected.

### Meta-analysis on patient databases

Oncomine and Gene expression Omnibus (GEO) databases were queried to identify the prognostic role of PRC2 components in PC. GEO database is available at http://www.pubmed.org (GEO profiles) and provides raw expression data from several gene expression arrays. Selected data compare PRC2 gene expression in normal prostate, primary tumor and metastasis. Data have been selected from Varambally et al. [26] because this study was an integrated molecular profiling of gene expression in PC samples. In this work, a significant concordance between EZH2 mRNA and protein was found. Oncomine database has been described in the previous section. In this case, it has been employed to investigate the correlation between PRC2 genes and PC prognosis. In addition, Oncomine provides curated sets of genes ("concepts"). Among these, "Polycomb group (PcG) target genes in embryonic stem cells" is a set of genes specifically silenced by PcG to maintain stem cell self-renewal. This concept was crossed with all Oncomine concepts derived from PC clinical analysis, in order to identify significant overlap; p-value was set at 0.01.

### Statistical Analysis

Unless otherwise specified, all experiments were done in triplicate and were repeated at least twice. Data were expressed as mean values SE or SD and were analyzed though GraphPad Prism software. The level of significance was set at p < 0.05.

## Results

### Pc Progression is Associated with Prc2 Gene Overexpression

To investigate the role of PRC2 gene silencing in PC progression, we queried the Oncomine database. One pre-defined Oncomine concept is "PcG target genes in human embryonic stem cells". This set includes 652 genes that were shown to be silenced by PRC2 through H3K27 methylation in embryonic stem cells [27]. Interestingly, we found that PRC2 target genes were specifically down-regulated in metastatic and high N stage PC. In addition, PRC2 target gene silencing predicted shorter overall survival and disease-free survival (Table 1).

**Table 1:** Prognostic role of PcG-target genes in PC samples

ASSOCIATED CONCEPT	P VALUE	ODDS RATIO
Dead at 3 Years Top 10% Under-expressed (Nakagawa Prostate 2)	5.65E-04	6.1

Recurrence at 5 Years - Top 5% Under-expressed (Holzbeierlein Prostate)	0.001	2.5

High Grade Top 1% Under-expressed (Bittner Prostate)	0.001	2.6

Advanced N Stage Top 10% Under-expressed (Nakagawa Prostate 2)	5.65E-04	6.1

Advanced Stage Top 5% Under-expressed (Dhanasekaran Prostate)	0.04	2.2

The Oncomine concept "PcG target genes in human embryonic stem cells" was crossed with all available prognostic data in PC samples (associated concepts). PcG target genes are significantly underexpressed in poor-prognosis, high stage PC. In brackets, Oncomine identification name.

**Figure 1:**
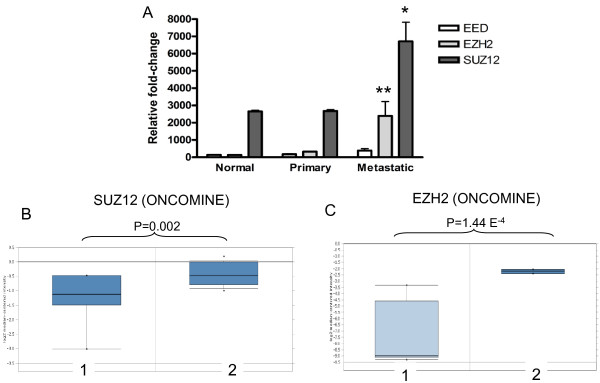
**PRC2 gene expression in PC patients**. A, comparison of PRC2 mRNA levels in normal prostate, primary and metastatic PC (GEO database). *p < 0.05, **P < 0.01. B, C: prognostic role of EZH2 and SUZ12 mRNA levels in PC sample (Oncomine database).

To confirm our findings, we queried the GEO database to compare PRC2 gene expression in normal prostate, primary tumors and metastatic samples. We found an increase of all PRC2 components with PC progression (Figure 1A). In particular, EZH2 and SUZ12 expression was significantly higher in metastatic PC, compared to primary tumors.

Finally, to confirm the negative prognostic role of PRC2 genes in PC, we queried PRC2 gene expression in the Oncomine database. As shown in Figure 1B and 1C, high EZH2 and high SUZ12 expression are positively correlated with metastatic spreading. These results confirm that PC progression is associated with increased PRC2 gene expression and PcG target gene silencing.

### Dznep Activity on Pc Cells and Prostatospheres

**Figure 2:**
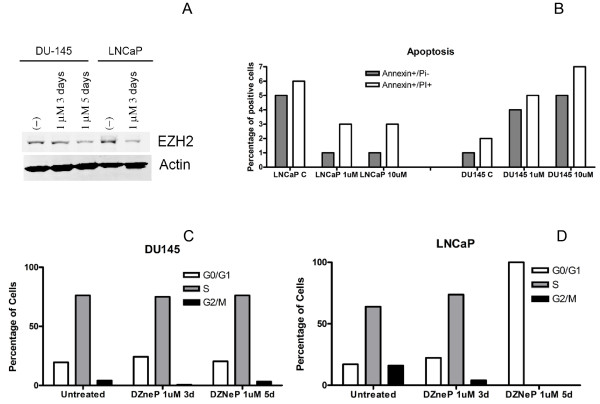
**Effects of DZNeP on PC cells**. A, Western blot analysis. (-), untreated cells. B, Annexin-PI staining on untreated and treated cells. Columns mean volume. C, D: cell cycle distribution after DZNeP treattment. Columns, mean volume. Cells were treated with 1 μM DZNeP for 3 and 5 days.

Due to the role of PRC2 genes in PC progression and prognosis, we tested the effect of the PRC2 inhibitor DZNeP on LNCaP and DU145 cells. We first confirmed that doses as low as 1 μM DZNeP were able to almost abolish EZH2 expression (Figure 2A) and reduced histone H3K27 trimethylation by 33% (quantification performed by LI-COR Odyssey IR Imaging System; Additional File 1). This is in line with the evidence that histone lysine methylation can be mediated by enzymes other than EZH2[28]. In LNCaP cells, EZH2 silencing was already evident after 3 days, while in DU145 cells we could find an effect after 5 days. Annexin-PI staining showed that DZNeP was able to trigger early and late apoptosis in DU145 cells (Figure 2B). We did not observe this effect on LNCaP cells. Cell cycle analysis showed that DZNeP treatment did not affect cell cycle distribution in DU145 cells, while inducing a consistent G0/G1 arrest in LNCaP cells (Figures 2C,D). For our experiments, we used DZNeP doses that showed anti-cancer activity, but were not harmful for non-transformed cells. In order to compare DZNeP activity with other commonly used epigenetic drugs, we treated LNCaP and DU145 cells with Trichostatin A (TSA) and 5-aza-2-deoxycitidine (5-aza) at doses non-toxic for normal cells[22,23]. As shown in Additional File 2, both drugs did not induce significant apoptosis in cancer cells.

**Figure 3:**
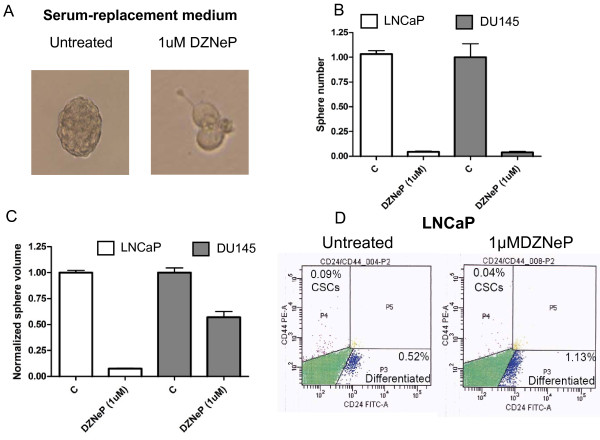
**Effects of DZNeP on PS and CSC markers**. A, representative picture of cells grown in SCM for 7 days. B, C PS number and volume in untreated and treated cells (1 μM, 7 days). Columns, mean volume; bars standard deviation. D, CSC marker expression. LNCaP cells were treated with DZNeP (1 μM, 3 days) and then sorted as described under "Flow cytometric analysis and separation".

Due to its effect on total cancer cells, we tested the hypothesis that 1 μM DZNeP was effective in inhibiting prostatosphere (PS) formation. We used a 1 week treatment in SCM, because PS form PC cell lines formed after 1 week culture in stem cell medium are enriched for CSCs and highly tumorigenic [8]. As shown in Figure 3, PS formation was completely abrogated by 1 μM DZNeP in both LNCaP and DU145 cells. Cells treated with DZNeP were generally unable to grow as spheres, and assumed a branched shape (Figure 3A). In both cell lines, sphere growth inhibition was higher than 95%. Mean sphere diameter was reduced more than 2-fold in DU145 cells, and almost undetectable in LNCaP cells. Interestingly, non-toxic doses of TSA and 5-aza were not able to eliminate PS formation (Additional File 3). In particular, 5-aza reduced sphere number by 50% in LNCaP cells, with weaker effect on DU145 cells. TSA eradicated PS formation in LNCaP cells (more than 95% inhibiton) but not in DU145 cells. Both drugs had weaker effects in comparison to DZNeP on PS diameter. These results show that DNZeP is effective in inhibiting PC cell growth in vitro, is selective for PC cells at doses higher than 1 μM, and abrogates PS self-renewal.

To further corroborate our findings, we investigated the effects of DZNeP treatment on CSC markers (CD44 and CD24). After DZNeP treatment (1 μM, 3d), LNCaP cells showed a decrease in the CD44^+^/24^- ^fraction, which is the CSC-enriched population (figure 3D). Interestingly, DZNeP treatment also increased the CD44^-^/24^+ ^fraction, which is represented by more differentiated cancer cells. Keeping with this observation, CD44^+^/24^- ^sorted cells were almost completely killed by the same DZNeP schedule (1 μM, 3d) (Additional File 4).

### Effects of Dznep on Pc Invasion and Tumorigenicity

**Figure 4:**
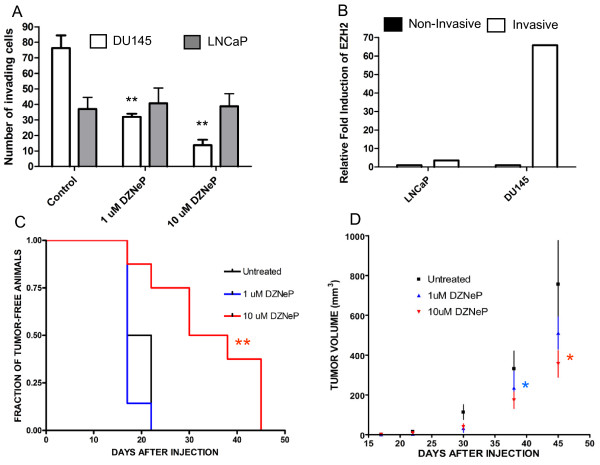
**Effects of DZNeP on invasion and *in vivo *tumor growth**. A, Cells were treated with DZNeP (1 μM, 3 days) and assayed for cell viability at the end of the treatment (trypan blue staining). Alive cells were used for Matrigel invasion assay, as described in "Materials and Methods". Columns, mean volume; bars, standard deviation. **p < 0.01 with respect to untreated cells (T test). B, mRNA levels in invading and non-invading cells (QT-PCR). C, effects of DZNeP on LNCaP cell tumorigenicity. **p < 0.01 (log rank test) with respect to untreated cells. D, effects of DZNeP on DU145 xenograft tumor volume. *p < 0.05 with respect to untreated cells (U test). Star colors refer to dose treatment (blue, 1 μM; red, 10 μM). Number of animals: untreated: 6; 1 and 10 μM: 8. Point, mean value; bar, standard deviation.

Since PRC2 genes seem to be involved in PC progression and metastatic spreading (Figure 1), we tested the hypothesis that DZNeP is able to inhibit *in vitro *invasiveness of PC cells. For this purpose, we treated PC cells with DZNeP, we then measured the concentration of viable cells through Trypan Blue staining, and then plated the same number of treated and untreated cells in a Matrigel invasion assay. This procedure discriminates between a general antitumor effect and a specific inhibition of invasion (Figure 4A). DU145 cells were generally more invasive than LNCaP cells. DZNeP significantly inhibited invasion in DU145 cells, but had no effect on LNCaP cells. To investigate the role of PRC2 genes in this process of invasion, we then compared EZH2 mRNA expression in invasive vs. non-invasive cells. EZH2 was markedly up-regulated after invasion (Figure 4B), however, EZH2 up-regulation was almost 20-fold higher in DU145 than in LNCaP cells. These results show that PRC2 genes are crucial for invasion in DU145, but not in LNCaP cells. To further investigate mechanisms of EMT inhibiton by DZNeP, we measured gene expression changes in 11 EMT-related genes. As shown in Additional File 5, DU145 cells treated with DZNeP showed more than 2-fold down-regulation of TGBR2 (TGF-β receptor 2) and SNAIL.

Finally, we tested the hypothesis that DZNeP treatment was able to impair tumorigenicty and tumor growth. For this purpose, we treated LNCaP and DU145 cells with 1 and 10 μM DZNeP (3 d) and then injected cells into immunocompromised NOD/SCID mice. For each cell line, the same number of viable treated and untreated cells was injected. We then measured time to palpable tumor formation and tumor volume for 45 days after injection. In LNCaP cells, 10 μM DZNeP was able to significantly inhibit time to tumor formation (Figure 4C), while in DU145 cells we did not observe this effect. However, DZNeP-treated cells formed tumors with a significantly slower growth rate, compared to untreated cells. For the 1 μM dose, we observed a significant difference in tumor volume for as long as 30 days after injection. For the 10 μM dose, this difference was still significant after 45 days (Figure 4D).

## Discussion

In the present work, we investigated the effects of the PRC2 inhibitor DZNeP on prostate CSCs, invasion and *in vivo *tumor growth. Our clinical data meta-analysis indicated that PRC2 genes are specifically silenced during PC progression, and that high expression of PRC2 genes is a negative prognostic factor in PC. These results confirm and broaden the already established role of EZH2 in PC progression[29]. In particular, we found that another member of PRC2 (SUZ12) is highly predictive of metastatic spreading, and that PRC2 target gene silencing is involved in PC progression, and associated with poorer survival. In addition, PRC2 genes are crucial for CSC self-renewal, and are thought to sustain PC growth [12] Thus, we tested the hypothesis that PRC2 inhibition affects PC tumorigenicity. We found several lines of evidence supporting this hypothesis. For our experiments, we employed 1 and 10 μM, 3d/5d-schedule, to make our results comparable to previously published data [19,20,30]. Doses as low as 1 μM DZNeP were able to inhibit PRC2-mediated H3K27 methylation, and displayed antitumor activity against PC cells (Figure 2). Interestingly, this schedule was shown to be non-toxic for normal cells [19]. In order to compare DZNeP to other epigenetic drugs, we tested PS formation in the presence of DNA-methyltransferase inhibitor 5-aza, and histone deacethylase inhibitor (HDACi) TSA. Non toxic concentrations of 5-aza did not affect PS formation. To the contrary, TSA eradicated PS in LNCaP, but not in DU145 cells (Additional File 3). These results suggest that DZNeP is more effective than other epigenetic drugs in eradicating PS. Interestingly, TSA seems to be more effective than 5-aza. Since HDACi were shown to indirectly target PRC2[31], we think that TSA effects on PS is in part mediated by PRC2 inhibition. Keeping with this hypothesis, it has been recently shown that 5-aza does not reactivate PRC2 targets in cancer cells[32].

In LNCaP cells, DZNeP treatment caused G0/G1 phase arrest, but was not able to trigger apoptosis. To the contrary, PRC2 targeting induced apoptosis in DU145 cells. This is interesting, since most antitumor treatments are generally more effective on LNCaP cells, which are thought to be a model for less aggressive and more chemosensitive PC [33]. Indeed, LNCaP cells express AR and a wild-type p53 protein, which are markers of early PC. In addition, DU145 cells are almost 100% CD44^+^, while the CSC fraction in LNCaP cells is less than 0.1% [4]. According to this proteomic profile, DU145 cells are more tumorigenic and more invasive than LNCaP cells [4,11]. Our results show that DZNeP specifically kills the CSC fraction in LNCaP cells (Figure 3D), while inducing apoptosis in all DU145 cells (Figure 2B), which are more "stem-like" cells. Interestingly, non-toxic concentration of other epigenetic drugs (TSA and 5-aza) were not able to induce apoptosis in both cell lines, suggesting that DZNeP may be peculiar among epigenetic modifers

We also found that DZNeP significantly inhibits invasion in DU145, but not in LNCaP cells (Figures 4A,B). A recent work showed that EZH2 gene silencing has different effects on PC cell lines [11]. In particular, EZH2 knockdown was less efficient in DU145 cells than other cell lines. Despite this, EZH2 silencing significantly reduced growth rate and invasion in these cells. To the contrary, EZH2 silencing on LNCaP cells had an effect on growth rate but not on invasiveness. The authors suggested that DU145 cells are particularly dependent on PRC2 function, like other AR-negative cells, and that the dependence on AR signaling decreases the dependence on EZH2 function.

Our data suggest that DZNeP inhibits SNAIL and TGFBR2, two master regulators of EMT in prostate cancer cells[34,35]. This is consistent with previous reports, showing that EZH2 mediates EMT through TGF-β pathway activation[14]. DZNeP seemed not to up-regulate some known EZH2 targets, like E-cadherin (CDH1). However, although CDH1 is silenced in some cancer cell lines by PRC2[15], it is still expressed by parental DU145 cells[36]. In these cell line, CDH1 is silenced only during migration and invasion[36]. Our experiments evaluated the effects of DZNeP on basal DU145 expression, because derivation of mRNA from invading cells is extremely difficult. Thus, it is not surprising to find that CDH1 is not up-regulated in our experimental conditions. It is likely that DZNeP hinders CDH1 silencing during DU145 cell invasion. SNAIL down-regulation may be a mechanism to maintain CDH1 expression, thereby inhibiting cell invasion. Indeed, PRC2 and SNAIL co-operate to inhibit CDH1 expression during EMT[37].

Our findings that DZNeP almost completely abrogated PS self-renewal, reduced CSC marker expression and impaired invasion through a possible EMT in DU145 cells is in line with our hypothesis, and show the potential of this drug for anti-metastatic and CSC-specific treatment. This is particularly interesting, since CSC are the seeds of metastatic spreading [5], and since PC metastasis are the main cause of cancer-related death [38].

*In vivo *injection of DZNeP pre-treated cells produced different results in LNCaP and DU145 cells (Figures 4C,D). In the less tumorigenic cell line (LNCaP), 10 μM DZNeP was able to significantly reduce tumor formation in NOD/SCID mice. 1 μM DZNeP had no effect on tumorigenicity. These results show that *in vivo *anti-CSC activity may require higher drug doses, that are however achievable and safe in animal models [18,39]. Similar results were obtained for the anti-CSC activity of temozolomide in glioblastoma [40]. In such a study, 500 μM temozolomide almost completely abrogated glioblastoma CSC clonogenicity *in vitro*. However, the same drug concentration did not reduce the percentage of Ki67-positive lesions formed after injection into immunocompromised mice. Interestingly, this result was obtained with a highly chemoresistant cell line (R28). Future clinical trials should demonstrate that a 10 μM DZNeP concentration is also clinically achievable and non-toxic in humans. In addition, it is worthy to note that we pre-treated cells with DZNeP and then injected alive cells. Thus, our results specifically investigate the anti-tumorigenic role of DZNeP, which is not dependent on its apoptotic activity on cancer cells [19]. In DU145 cells, DZNeP pre-treatment did not affect tumor formation, but significantly inhibited tumor growth. DZNeP-treated cells formed significantly smaller tumors after as many as 30 (1 μM) and 45 (10 μM) days post-injection. Since DU145 cells are more stem-like and more dependent on PRC2 function, we think that these cells can escape from DZNeP antitumorigenic activity. Nonetheless, we found a long-lasting effect on tumor growth. Our results may support the idea that DZNeP is more active to prevent PC metastasis than to treat local advanced PC.

In conclusion, we showed that DZNeP is able to impair CSC self-renewal and *in vivo *tumor development at doses that are safe in mouse models. This results can pave the way to the investigation of DZNeP anticancer activity in PC patients.

## Competing interests

The authors declare that they have no competing interests.

## Authors' contributions

FC conceived the study and performed Oncomine, GEO analysis and prostatosphere experiments. In addition, he wrote the paper. EMH performed in vivo experiments, western blots, cell cycle and apoptosis analysis. In addition, she critically revised the paper. LAM performed invasion experiments, and EMT gene expression profiling, and gave insights into EMT-CSC relationship. SMC and LS designed and performed experiments with 5-AZA and TSA. VEM supported experimental design with his knowledge on DZNeP and critically revised the paper. RD critically revised the paper. WLF participated in experimental design and critically revised the paper. All authors read and approved the final manuscript.

## Supplementary Material

Additional File 1 **H3K27 trimethylation in LNCaP cells**.: Western blot data comparing untreated cells, and cells treated with DZNeP (5 μM, 3d). Click here for file

Additional File 2 **Annexin-PI staining of LNCaP (A) and DU145 (B) cells treated with 5-AZA and TSA**. Cells were treated at concentration shown to be non-toxic for normal cells, as described in "Materials and Methods". Experiment were repeated twice. Here is shown one representative experiment. Click here for file

Additional File 3 **PS number (A, C) and PS diameter (B, D) after treatment with TSA and 5-AZA**. Cells were treated at concentration shown to be non-toxic for normal cells, as described in "Materials and Methods". Experiment were repeated twice. Here is shown one representative experiment. Click here for file

Additional File 4 **effects of DZNeP on CD44**^**+**^**/24**^**+ **^**cells**. LNCaP cells were sorted as described under ""Flow cytometric analysis and separation". Cells were treated with 1 μM DZNeP (5 days) and cell viability was assessed trough "Cell Titer Glo" assay (Promega). Click here for file

Additional File 5 **Gene expression changes in EMT-related genes induced by DZNeP**. DU145 cells were treated with DZNeP (10 μM, 3d). Fold change refers to relative mRNA levels, measured as described in "Materials and Methods". Click here for file
