# Supplementary figures and images for: Pharmacologic disruption of Polycomb Repressive Complex 2 inhibits tumorigenicity and tumor progression in prostate cancer
Source: Mol Cancer. 2011 Apr 18;10:40. doi: 10.1186/1476-4598-10-40 (PMC3100246; doi:10.1186/1476-4598-10-40)

## Slide 1
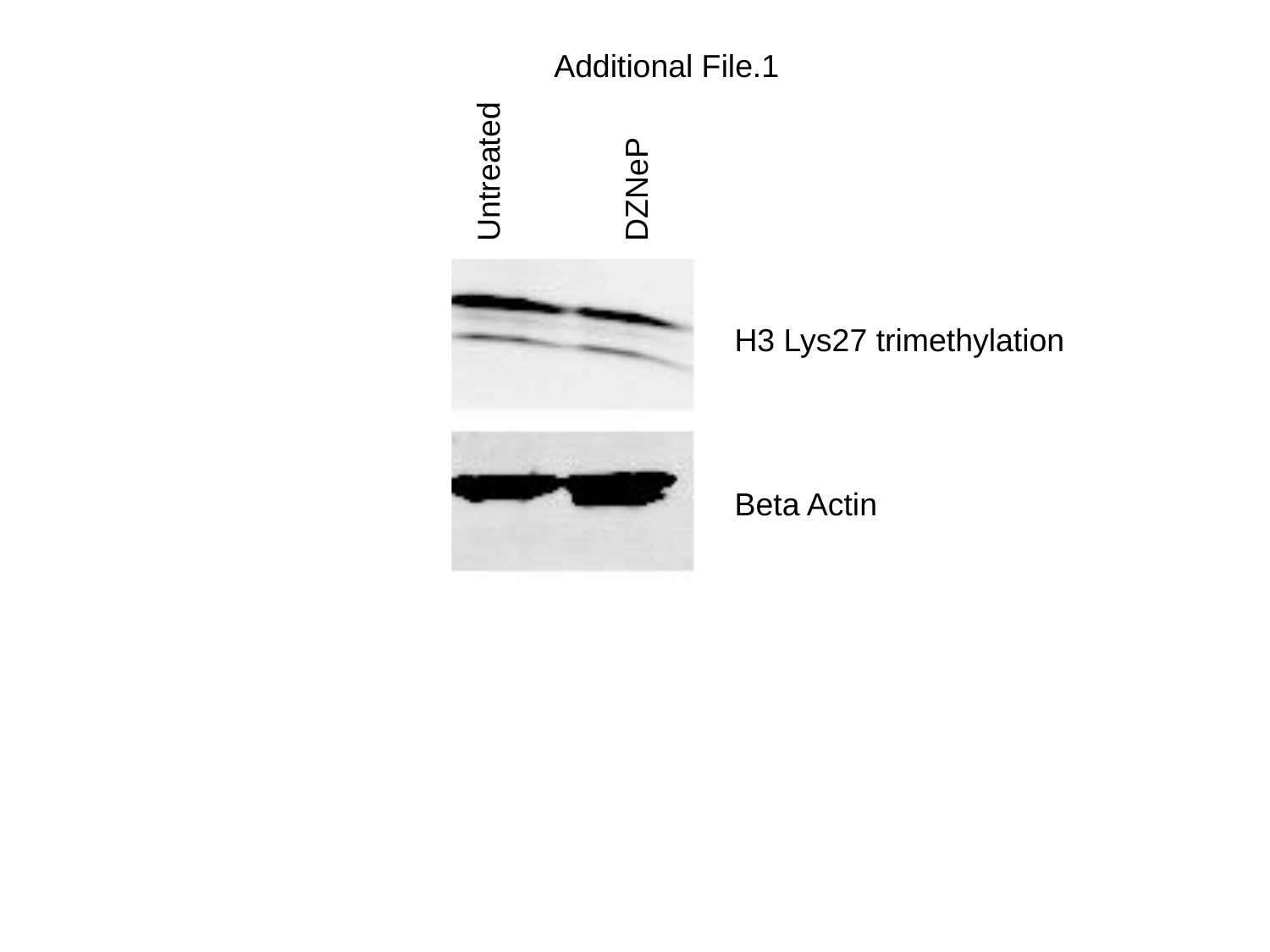

Additional File.1
Untreated
DZNeP
H3 Lys27 trimethylation
Beta Actin

Supplement: Additional File 1 — H3K27 trimethylation in LNCaP cells.: Western blot data comparing untreated cells, and cells treated with DZNeP (5 μM, 3d). [file 1476-4598-10-40-S1.PPT]

## Slide 1
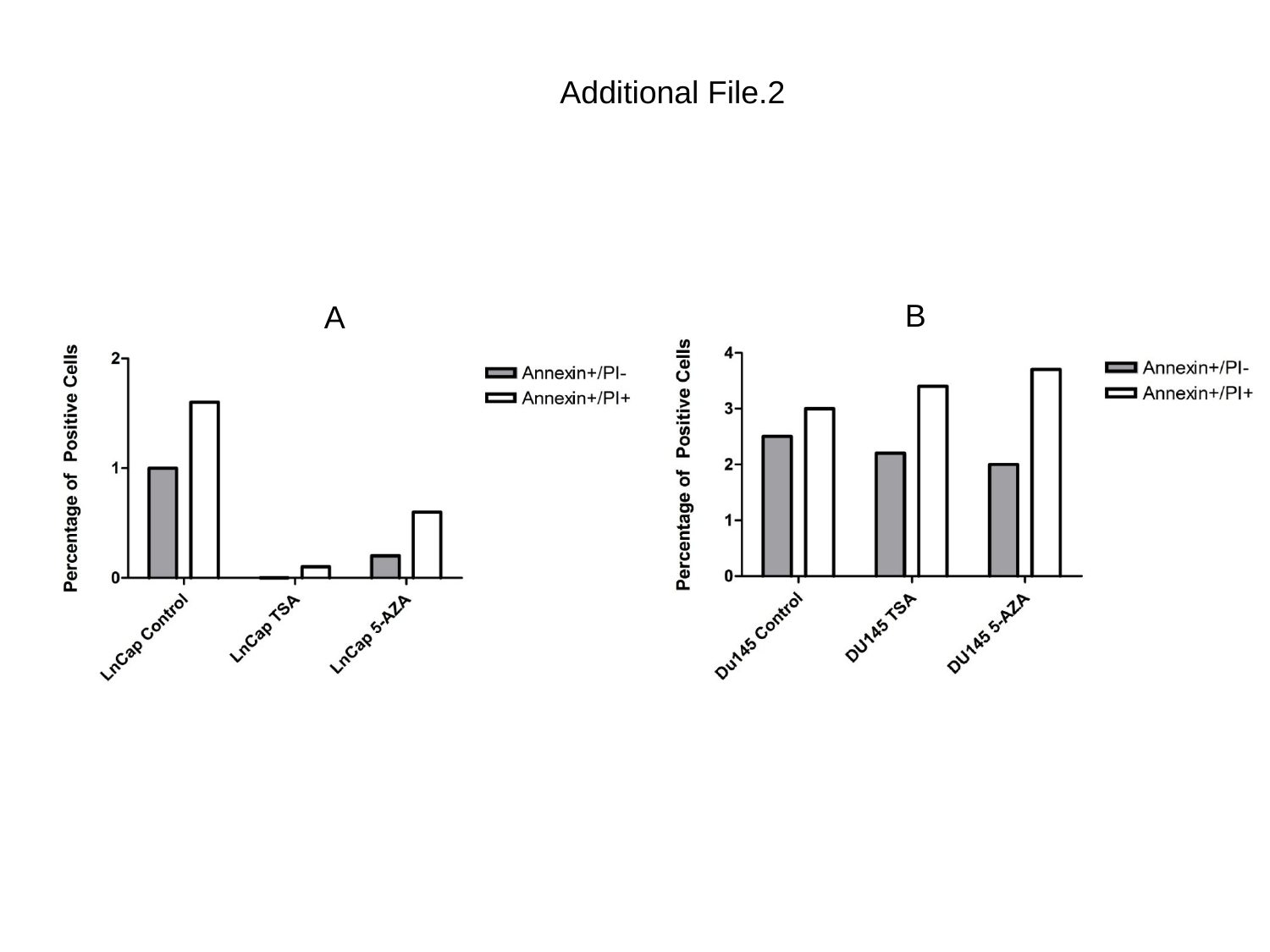

Additional File.2
B
A

Supplement: Additional File 2 — Annexin-PI staining of LNCaP (A) and DU145 (B) cells treated with 5-AZA and TSA. Cells were treated at concentration shown to be non-toxic for normal cells, as described in "Materials and Methods". Experiment were repeated twice. Here is shown one representative experiment. [file 1476-4598-10-40-S2.PPT]

## Slide 1
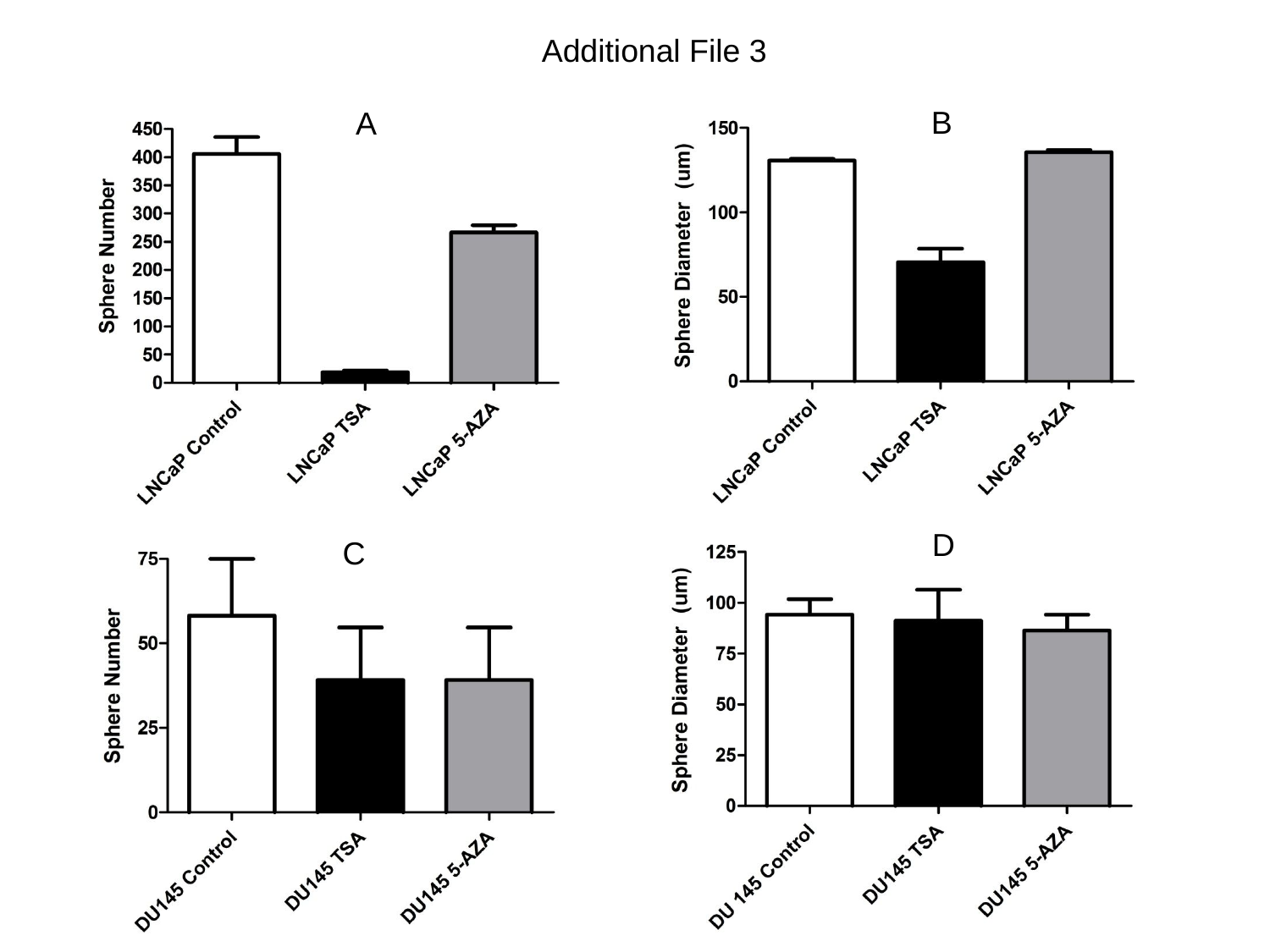

Additional File 3
B
A
D
C

Supplement: Additional File 3 — PS number (A, C) and PS diameter (B, D) after treatment with TSA and 5-AZA. Cells were treated at concentration shown to be non-toxic for normal cells, as described in "Materials and Methods". Experiment were repeated twice. Here is shown one representative experiment. [file 1476-4598-10-40-S3.PPT]

## Slide 1
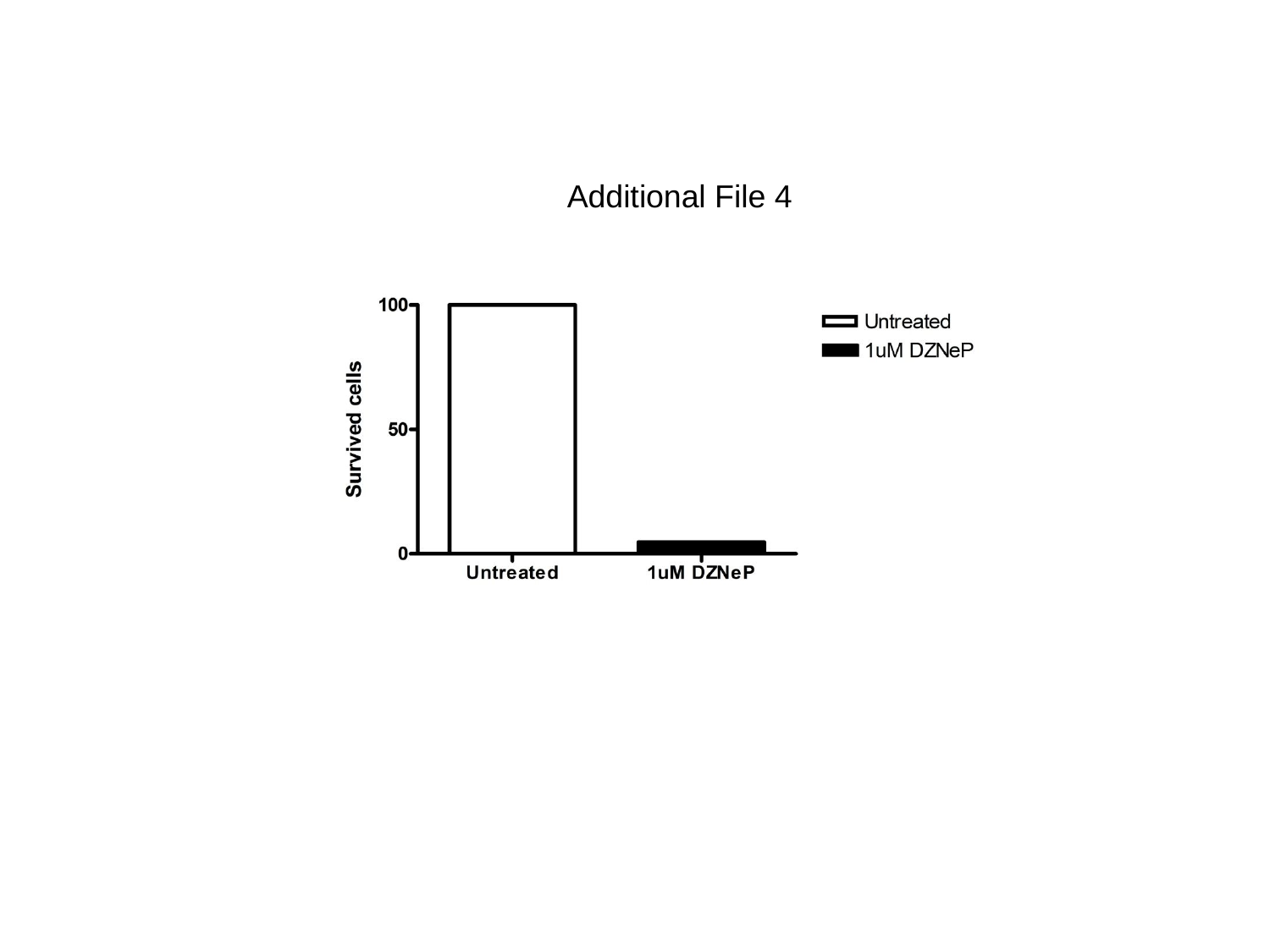

Additional File 4

Supplement: Additional File 4 — effects of DZNeP on CD44+/24+ cells. LNCaP cells were sorted as described under ""Flow cytometric analysis and separation". Cells were treated with 1 μM DZNeP (5 days) and cell viability was assessed trough "Cell Titer Glo" assay (Promega). [file 1476-4598-10-40-S4.PPT]

## Slide 1
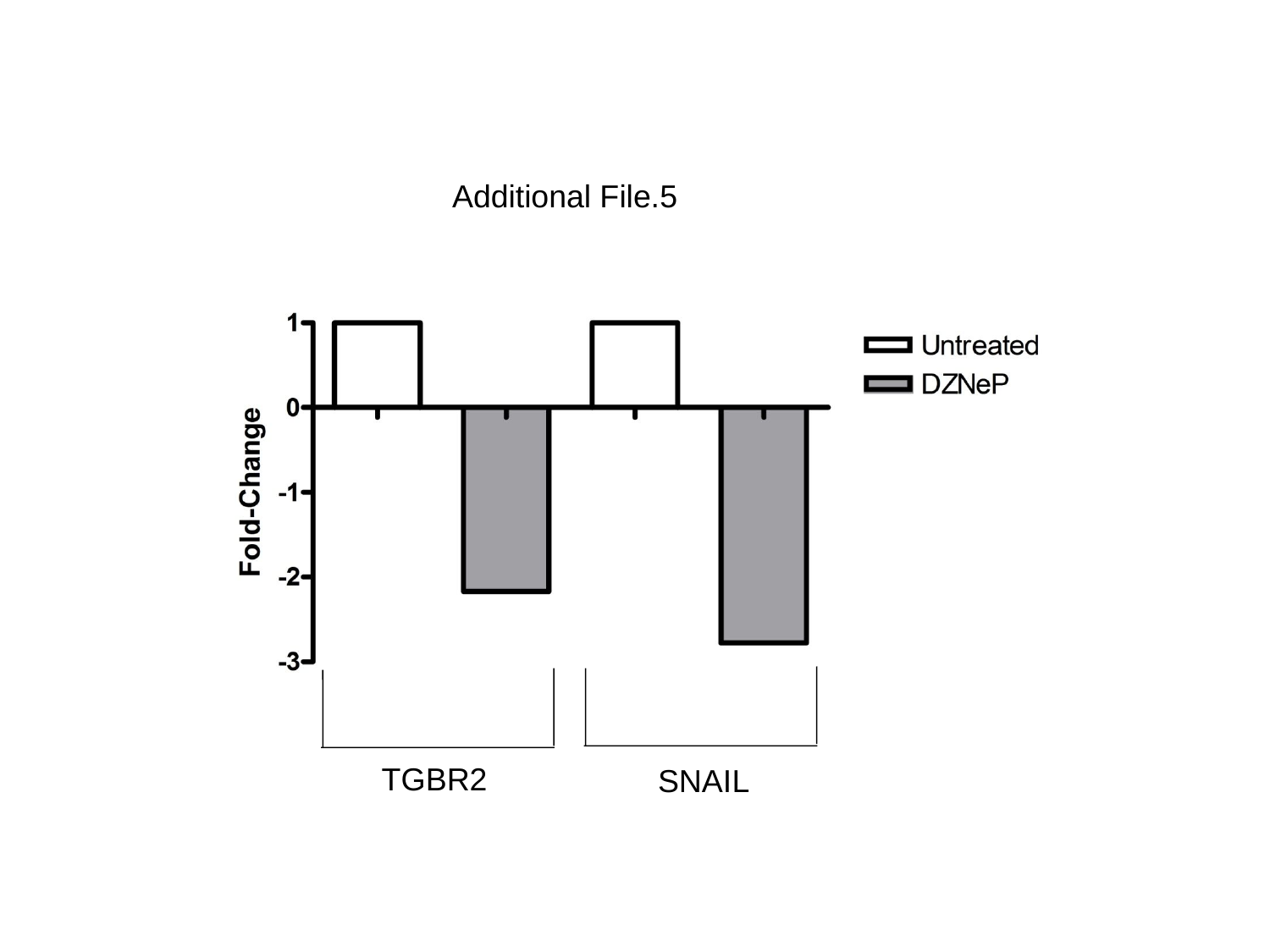

Additional File.5
TGBR2
SNAIL

Supplement: Additional File 5 — Gene expression changes in EMT-related genes induced by DZNeP. DU145 cells were treated with DZNeP (10 μM, 3d). Fold change refers to relative mRNA levels, measured as described in "Materials and Methods". [file 1476-4598-10-40-S5.PPT]
